# Escher-Trace: a web application for pathway-based visualization of stable isotope tracing data

**DOI:** 10.1186/s12859-020-03632-0

**Published:** 2020-07-10

**Authors:** Avi Kumar, Jack Mitchener, Zachary A. King, Christian M. Metallo

**Affiliations:** 1 Department of Bioengineering, University of California, San Diego, 9500 Gilman Drive, La Jolla, CA 92093 USA; 2 Moores Cancer Center, University of California, San Diego, La Jolla, CA USA

**Keywords:** Stable isotope tracing, Metabolism, Escher, Visualization, Web application

## Abstract

**Background:**

Stable isotope tracing has become an invaluable tool for probing the metabolism of biological systems. However, data analysis and visualization from metabolic tracing studies often involve multiple software packages and lack pathway architecture. A deep understanding of the metabolic contexts from such datasets is required for biological interpretation. Currently, there is no single software package that allows researchers to analyze and integrate stable isotope tracing data into annotated or custom-built metabolic networks.

**Results:**

We built a standalone web-based software, Escher-Trace, for analyzing tracing data and communicating results. Escher-Trace allows users to upload baseline corrected mass spectrometer (MS) tracing data and correct for natural isotope abundance, generate publication quality graphs of metabolite labeling, and present data in the context of annotated metabolic pathways. Here we provide a detailed walk-through of how to incorporate and visualize ^13^C metabolic tracing data into the Escher-Trace platform.

**Conclusions:**

Escher-Trace is an open-source software for analysis and interpretation of stable isotope tracing data and is available at https://escher-trace.github.io/.

## Background

Metabolism plays a central role in all areas of biology. Metabolic reprogramming at the cellular level has been implicated in numerous diseases ranging from diabetes [1, 2] to cancer [3, 4]. Understanding metabolic phenotypes involves not only analyzing metabolite abundances (i.e., metabolomics) but also changes in metabolic pathway activity or flux. Stable isotope tracing studies are an effective method for investigating intracellular metabolic fluxes [5, 6] that have been increasingly used in the last decade [7]. Insight into pathway activity can be gleaned by applying a stable isotope labeled metabolite to a cell or in vivo system and evaluating where the heavy isotopes are transferred. In recent years, tracing studies have been critical for identifying alterations in tricarboxylic acid [8, 9], serine [10], branched chain amino acids [11, 12], and NAD(P)H metabolism [13–15] in a variety of cell and organ systems. Stable isotope tracing experiments utilizing a single tracer and nominal resolution GC-MS analytics in particular have become widely adopted due to their reliability, low cost, and the wealth of information they can provide.

Tracing data sets are initially presented in large data tables which contain the labeling patterns (i.e. isotopologue or mass isotopomer distributions) of all measured compounds in each sample. Interpreting such datasets is best accomplished in the context of metabolic networks and each metabolite’s location within metabolic pathways. Additionally, proper reporting of data requires extensive plotting of results, which can be normalized and presented in various ways. The discovery process, which is typically iterative, can be extremely time consuming when dealing with many metabolites or samples. As such, a data visualization platform where isotopologue distributions and related data can be presented graphically in the context of metabolic networks would be very beneficial to the research community. Such a platform would contextualize the data and remove tedious intermediary steps allowing researchers to better focus on validating and interpreting the meaning of their results.

Although numerous software packages are available to analyze metabolomics data, few include capabilities for interpreting stable isotope labeling. XCMS [16], OPENMS [17], as well as vendor-specific software packages allow for integration of mass spectrometry data to quantify metabolite abundances but do not provide options for analyzing metabolite labeling. IsoMetlin [18] allows for identification of isotopically labeled compounds from MS and MS/MS spectra, while software packages such as IsoCor [19], ICT [20], ElemCor [21], and IsoCorrectoR [22] allow users to correct isotopic labeling for natural isotope abundance. MAVEN allows for quantitation of high-resolution metabolite labeling as well as natural isotope correction but is not designed for widely available GC-MS systems [23]. Metaboanalyst [24] and Omix [25] allow for visualization of metabolomic and fluxomic datasets in the context of a network but do not provide options for processing or overlaying tracing data. As a result, scientists running tracing studies are required to use multiple software packages to correct their data for natural isotope abundance, perform analysis, prepare figures, and communicate their results.

The most effective way to understand data from tracing experiments is to view metabolite labeling patterns, enrichments, and abundances together on top of a metabolic map. Escher-Trace fills this need by allowing users to overlay multiple types of tracing data on top of Escher metabolic network maps. The software has an interactive, user-friendly, graphical user interface, has a low entry level (i.e. is accessible to users with little tracing experience), and is specifically catered toward users running single tracer studies with GC-MS analytics. Escher-Trace enables users to correct for natural isotope abundance, generate publication quality graphs of metabolite labeling, and present data in the context of pathways. Escher is compatible with BiGG Models [26], providing access to a set of metabolic maps, and the Escher platform provides a comprehensive library of metabolites and metabolic reactions that can be used to seamlessly generate new maps. The ability to generate graphs of multiple data types and compatibility with Escher make Escher-Trace a powerful tool for analyzing and visualizing tracing datasets. We walk through a specific use case of how one can employ Escher-Trace to analyze a stable isotope traced data set and generate a figure summarizing the primary findings of the experiment.

### Implementation

**Fig. 1:**
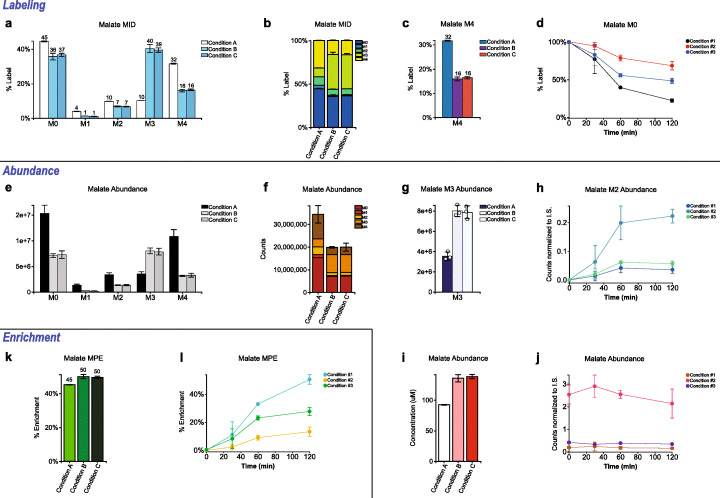
Graph types available in Escher-Trace. **a** Mass isotopomer distribution. **b** Stacked mass isotopomer distribution. **c** Single isotopologue label. **d** Kinetic single isotopologue label*. **e** Abundance. **f** Stacked abundance. **g** Single isotopologue abundance**. **h** Kinetic single isotopologue abundance*. **i** Quantitative abundance***. **j** Kinetic total abundance*. **k** Enrichment****. **l** Kinetic enrichment* ****. (*) Requires time point information to be input using Analysis ➔ Enter Time Points in the Escher-Trace Menu. (**) Shown with individual data points plotted, accessed by setting Graph Attributes ➔ Plot Individual Values to ON. (***) Requires quantitative standard information to be input using Analysis ➔ Enter Quantitative Standards in the Escher-Trace Menu. (****) Requires element count of tracer of interest to be input using Data Displayed ➔ Isotopologues to Display in the Escher-Trace Menu. Note: All data used to make graphs in this figure were simulated

Escher-Trace is built on top of the Escher [27] interface, a web based metabolic pathway visualization platform, using javascript and the D3.js library. Escher-Trace allows users to upload stable isotope labeled metabolomic data sets into Escher by clicking the “Import Tracing Data” button in the bottom right hand corner of the screen. Data files can be uploaded in CSV format as either baseline corrected (Additional file 1) or both baseline and natural isotope abundance corrected mass spectrometer counts (Additional file 2) or alternatively in JSON format if reloading a prior Escher-Trace workspace. The required format of uploaded data is specified in the user documentation (https://escher-trace.readthedocs.io/). If uploading data that is not corrected for natural isotope abundance, the user will be asked to indicate which type of tracer was used in their data set (e.g. 13C, 15 N, 2H). When uploading data for the first time, the user will be instructed to organize their samples into groups based on experimental conditions. Sample data within the same group will be averaged together and presented with standard deviation in graphs. Data sets with over one hundred samples organized into over forty groups have successfully been uploaded to the tool. However, the largest default color scheme in Escher-Trace contains fifteen color entries, if more groups are included, the user can create and utilize larger color schemes by selecting **Graph Attribute ➔ Color Scheme** from the Escher-Trace menu, additional instruction can be found in the user documentation. After organizing sample data files into groups, isotopologue distributions which correspond to metabolites included in the Escher map will be displayed next to the corresponding Escher metabolite node. Data is mapped by connecting the BiGG IDs of metabolites entered by the user to the corresponding Escher nodes. BiGG IDs are standardized identifiers of metabolites included in the BiGG Models database [26], which allows for the connection of Escher map nodes to genome scale metabolic models and external databases such as KEGG [28] and BioCyc [29]. All unmapped metabolite data is displayed on the left-hand side of the map. Data that is uncorrected for natural isotope abundance is corrected using the user selected tracer type, metabolite formula, actual measurements, natural stable isotope enrichment information, and the algorithm presented by Fernandez et al. [30]. Matrix calculations are performed using functions from the math.js library. The correction algorithm used by Escher-Trace is best suited to correct small molecule metabolite data generated by a nominal resolution mass spectrometer. Data which does not fit these conditions can still be visualized in Escher-Trace, but must be corrected first using a separate correction software and uploaded as a corrected csv data file (the format for which can be found in the user documentation). A comparison of results obtained from the correction algorithms of Escher-Trace and IsoCor can be found in Additional file 3. Escher-Trace can visualize metabolite isotope labeling, enrichment, and abundance as single bar or stacked bar plots for steady state labeling studies or as line graphs for time-course/kinetic studies (Fig. 1). Isotopologue distributions are calculated by dividing the abundance of each isotopologue by the sum total of all isotopologues of the metabolite.

The user can interact with their data by two means (1) using the Escher-Trace Menu located on the right-hand side of the screen or (2) right-clicking individual graphs. With the Escher-Trace menu, the user can make changes which affect graphs across the network such as altering data types or metabolites that are visualized, normalizing abundances of metabolites, and saving the Escher-Trace workspace in JSON format. By right-clicking individual graphs, the user can download the selected graph in SVG or PNG format, remove the graph from the map, view the data used to generate the graph, or generate additional graphs relating to the specific metabolite including data for additional fragments.

## Results and discussion

To demonstrate a typical use case of Escher-Trace, we will analyze data from the Huh7 hepatocellular carcinoma cell line grown with [U-^13^C_5_]glutamine in normoxic (21% oxygen) and hypoxic (1% oxygen) conditions. Hypoxia induces a classical metabolic reprogramming phenotype in highly proliferative cells [31]. We will walk through how a user might identify reprogramming induced by hypoxia and generate a figure that communicates this phenotype with Escher-Trace.

**Fig. 2:**
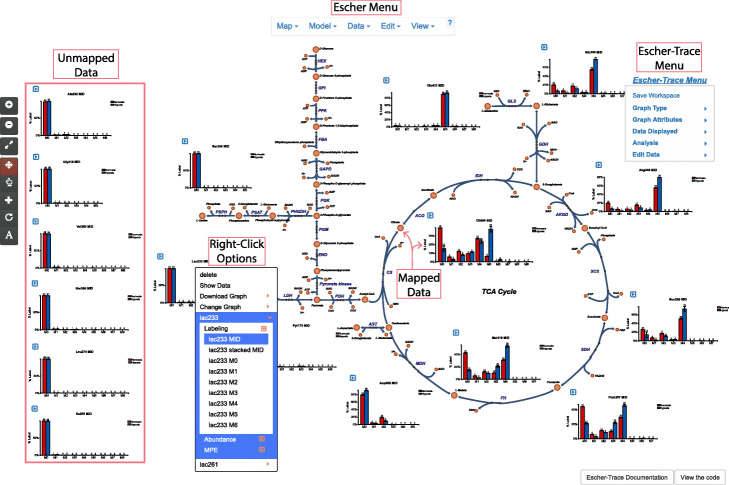
Escher-Trace Interface. The Escher-Trace menu can be used to cycle through graphs of labeling, abundance, and enrichment, alter graph aesthetics, normalize abundances, reorganize data and data files, save the Escher-Trace workspace and more. Data is mapped to Escher metabolite nodes by BiGG ID. Graphs of unmapped metabolites are included on the left-hand side of the Escher canvas. All graphs can be right-clicked to access additional graph types for the selected metabolite as well as graph specific functions. The Escher menu and all of its functionality related to map editing and data overlay is accessible when using Escher-Trace

First, we must upload our raw mass spectrometer counts CSV file (available as Additional file 1) which were analyzed via nominal resolution gas chromatography-mass spectrometry (GC-MS) and integrated with an available MS integration software package. The data is baseline corrected but remains uncorrected for natural isotope abundance, so we upload the CSV file to Escher-Trace as an uncorrected CSV file and select the tracer type as “^13^C”. We organize the sample data files into two distinct groups which are named “Normoxia” and “Hypoxia” to represent each experimental condition. After submitting the sample order, isotopologue distributions of our data appear on top of the Escher map (Fig. 2). At first glance, one can identify that our experimental conditions produce distinct labeling patterns in TCA cycle intermediates.

**Fig. 3:**
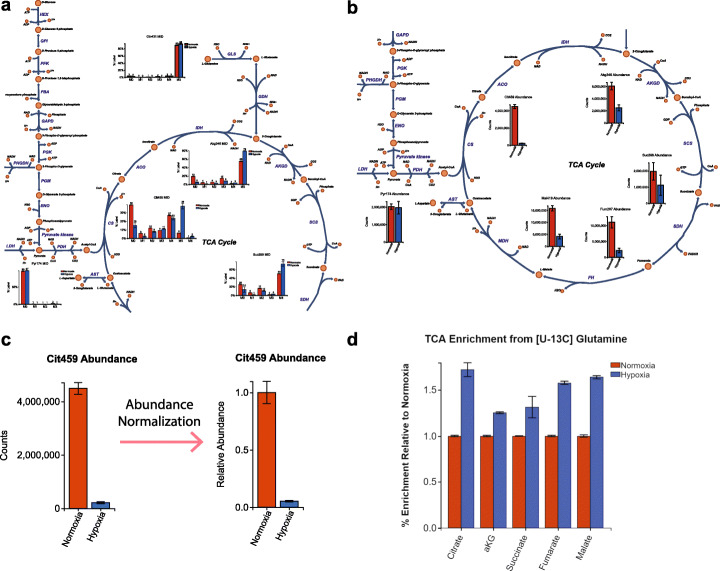
Data Analysis. **a** TCA intermediates labeling from a [U-^*13*^C_5_]glutamine tracer, generated after the initial submission of data to Escher-Trace. **b** TCA intermediate abundance generated by selecting Graph Type ➔ Total Abundance from the Escher-Trace menu. **c** Citrate abundance before and after entry of abundance normalization information via Analysis ➔ Normalize Abundance from the Escher-Trace menu. **d** TCA enrichment from a [U-^*13*^C_5_]glutamine tracer, generated by selecting Analysis ➔ Compare Metabolites from the Escher-Trace menu

Upon closer observation of the isotopologue distributions for TCA intermediates, we can identify a unique labeling distribution on citrate. Specifically, we observe increased relative abundance of M5 citrate label (over 6x increased) in hypoxia compared to normoxia (Fig. 3a). The number of isotopologues visualized in Escher-Trace graphs can be adjusted for clarity by selecting **Data Displayed ➔ Isotopologues to Display** from the Escher-Trace menu and reducing the isotopologue limit for the metabolite of interest. Using the metabolic map, we can identify that M5 citrate can only be generated reductively (in the counter-clockwise direction) from alpha-ketoglutarate (aKG). If aKG is oxidized in the clockwise direction of the TCA cycle, M4 isotopologues of the remaining TCA intermediates will form. This includes M4 citrate which is generated from M4 oxaloacetate and unlabeled mitochondrial acetyl-CoA derived predominantly from unlabeled glucose-derived pyruvate. Increased M5 citrate formation is now known to be demonstrative of upregulated reductive carboxylation flux which has been observed in highly proliferative cells cultured in hypoxia [32].

To get a broader overview of how metabolite abundances are altered between the experimental conditions we select on the Escher-Trace menu **Graph Type ➔ Total Abundance**. This causes all metabolite data to switch from isotopologue distributions to abundances (Fig. 3b). We see that there appears to be a distinct decrease in the raw counts of TCA intermediates in cells under hypoxia. However, to properly analyze how intracellular abundances are impacted by hypoxia, we need to normalize our data based on our experimental workflow. Metabolite abundance normalization is performed by selecting **Analysis ➔ Normalize Abundances** on the Escher-Trace menu. Here a norvaline internal standard was spiked into all samples, so we select nor260, one of the two norvaline fragments we detect with our GC-MS method, for normalization of all metabolites. Additionally, cell count data can be entered to obtain per cell quantitation of metabolites (normoxia: 638333 cells, hypoxia: 668750 cells). We can also optionally select an experimental condition to normalize our data to simplify data presentation; in our case we present data relative to normoxia. After submitting our selections, total abundance graphs are rescaled (Fig. 3c). One can see that the per cell abundances of TCA intermediates are decreased in hypoxic conditions, as previously noted [32].

We can streamline data communication by producing graphs of data across metabolites. These types of graphs can by generated in Escher-Trace by selecting **Analysis ➔ Compare Metabolites** in the Escher-Trace menu. This option allows the user to select metabolites, experimental conditions, and data types (metabolite abundance, enrichment, or individual isotopologue labeling) to include in a bar graph. Using this feature, we generated a summary figure highlighting increased TCA intermediate enrichment from [U-^13^C_5_]glutamine in hypoxia (Fig. 3d).

**Fig. 4:**
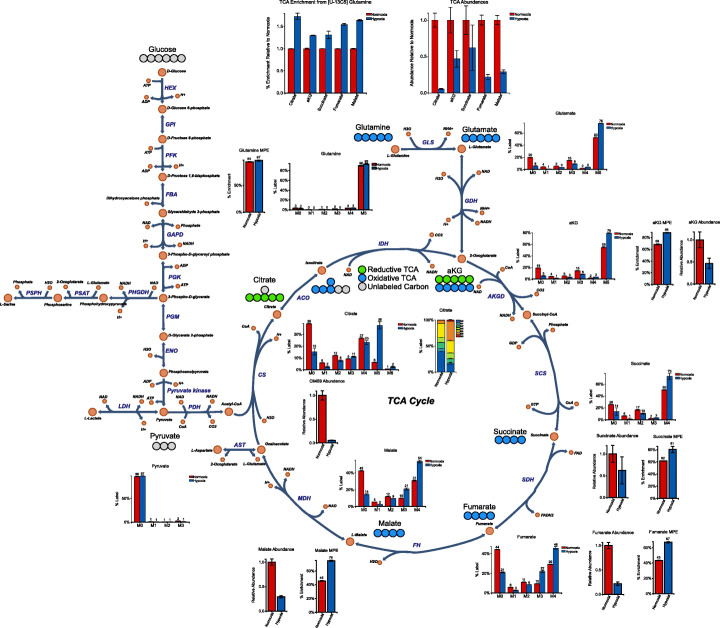
Complete ^13^C Figure. A complete ^*13*^C data figure showcasing the reprogramming of glutamine catabolism in Huh7 hepatocellular carcinoma cells grown in hypoxia compared to normoxia. Carbon circle diagrams were added by selecting Data Displayed ➔ Create Carbon Diagram from the Escher-Trace menu

Finally, to simplify the data presented on the metabolic map, we can select to only display data for metabolites of interest, glycolytic and TCA intermediates in this case, by using **Data Displayed ➔ Metabolites to Display** in the Escher-Trace menu. Figures of additional data types, including individual isotopologue labeling, metabolite abundance, and mole percent enrichment, as well as additional plot types (single or stacked bar plots) can be accessed using the context menu, by **right-clicking** any Escher-Trace graph, selecting a **metabolite fragment**, and then selecting the **data and plot type**. The title and y-axis labels of all graphs can be edited by clicking on them and entering in new text as needed for clarity. Finally, labeling diagrams can be introduced using **Data Displayed ➔ Create Carbon Diagram**, to generate the final figure (Fig. 4) This publication-quality figure can be downloaded as an SVG or PNG file using the Escher menu selecting **Map ➔ Export as SVG or PNG**. Individual graphs can be independently downloaded by **right-clicking** them and selecting **Download ➔ SVG or PNG**. The Escher-Trace workspace itself can be downloaded by selecting **Save Workspace** from the Escher-Trace menu. This workspace file can be sent to collaborators and reloaded in Escher-Trace to communicate findings or perform further analyses.

## Conclusions

Escher-Trace is a web-based software for visualizing and interpreting stable isotope tracing data in the context of a metabolic network. We have shown that Escher-Trace can facilitate calculation and presentation of labeling patterns resulting from alterations in pathway activity (i.e. hypoxia) and overlay results on an Escher pathway map. By providing a tool which allows users to correct for natural isotope abundance, visualize labeling patterns, and generate scientific figures all through a simple graphical user interface, we believe Escher-Trace is a significant addition to the software available to metabolic researchers.

### Availability and requirements

- **Project name:** Escher-Trace.
- **Project home page:**http://escher-trace.github.io
- **Operating system(s):** Platform independent.
- **Programming language:** HTML and Javascript.
- **License:** MIT License.
- **Any restrictions to use by non-academics:** none.

## Supplementary information

**Additional file 1. Escher-Trace Uncorrected Example Data File.csv** This file contains the data set used to generate all graphs in the manuscript. Data was generated by culturing Huh7 hepatoma cells, provided by M. Hemann, in normoxia or hypoxic (1% oxygen) conditions for 48 h with Dulbecco’s Modified Eagle Media containing 4 mM [U-^13^C_5_]glutamine. This file can be uploaded to Escher-Trace as described in the Implementation section. The data set is also available at https://escher-trace.readthedocs.io/.

**Additional file 2. Escher-Trace Corrected Example Data File.csv** This file contains the same data set as Additional file 1, however the data has been pre-corrected for natural isotope abundance. This file is also available in the Escher-Trace documentation (https://escher-trace.readthedocs.io/) where a walkthrough of how the file needs to be constructed and uploaded to Escher-Trace can be found.

**Additional file 3. Comparison of Escher-Trace and IsoCor NAC.docx** This file contains a comparison of stable isotope correction results obtained from Escher-Trace and IsoCor.

## Data Availability

All data generated or analyzed during this study are included in this published article [and its supplementary information files]. These files can also be accessed on the Escher-Trace version 1.1 site at https://www.escher-trace.github.io/. Escher-Trace documentation, which has in-depth walkthroughs of all Escher-Trace functionality, can be found at https://escher-trace.readthedocs.io/. The source code of Escher-Trace can be found at https://github.com/escher-trace/escher-trace.github.io.

## Abbreviations

a-KG: Alpha-Ketoglutarate
CSV: Comma-separated values
GC-MS: Gas Chromatography Mass Spectrometry
JSON: JavaScript Object Notation
MS: Mass Spectrometer
PNG: Portable Network Graphics
SVG: Scalable Vector Graphics
TCA: Tri-Carboxylic Acid
