# Supplementary material for: Escher-Trace: a web application for pathway-based visualization of stable isotope tracing data
Source: BMC Bioinformatics. 2020 Jul 10;21:297. doi: 10.1186/s12859-020-03632-0 (PMC7350651; doi:10.1186/s12859-020-03632-0)
Supplement: Supplementary file 3 — Additional file 3. Comparison of Escher-Trace and IsoCor NAC.docx This file contains a comparison of stable isotope correction results obtained from Escher-Trace and IsoCor. [file 12859_2020_3632_MOESM3_ESM.docx]

| Metabolite | Formula | Isotopologue | Counts | IsoCor Relative Abundance | Escher-Trace Relative Abundance |
| --- | --- | --- | --- | --- | --- |
| Citrate | C20H39O6Si3 | cit459(M0) | 22000 | 15.4% | 16.5% |
|  |  | cit460(M1) | 11300 | 3.1% | 2.2% |
|  |  | cit461(M2) | 15600 | 7.5% | 7.7% |
|  |  | cit462(M3) | 21200 | 11.5% | 11.5% |
|  |  | cit463(M4) | 37300 | 21.1% | 21.2% |
|  |  | cit464(M5) | 67900 | 38.8% | 38.8% |
|  |  | cit465(M6) | 26200 | 2.5% | 2.0% |
|  |  | cit466(M7) | 11400 | * | 0.3% |
|  |  | cit467(M8) | 2390 | * | -0.3% |
|  |  | cit468(M9) | 862 | * | 0.2% |
|  |  |  |  |  |  |
| a-ketoglutarate | C14H28O5N1Si2 | akg346(M0) | 896000 | 17.7% | 18.7% |
|  |  | akg347(M1) | 451874 | 5.3% | 4.4% |
|  |  | akg348(M2) | 408063 | 5.3% | 5.2% |
|  |  | akg349(M3) | 801000 | 14.0% | 14.1% |
|  |  | akg350(M4) | 299059 | 2.4% | 2.1% |
|  |  | akg351(M5) | 2890000 | 55.2% | 55.3% |
|  |  | akg352(M6) | 610000 | * | 0.0% |
|  |  | akg353(M7) | 281000 | * | 0.2% |
|  |  | akg354(M8) | 42200 | * | 0.0% |
|  |  |  |  |  |  |
| Aspartate | C18H40O4N1Si3 | asp418(M0) | 2080000 | 42.9% | 45.0% |
|  |  | asp419(M1) | 980000 | 6.8% | 4.9% |
|  |  | asp420(M2) | 951000 | 10.9% | 11.0% |
|  |  | asp421(M3) | 743519 | 9.5% | 9.3% |
|  |  | asp422(M4) | 1710000 | 30.0% | 30.0% |
|  |  | asp423(M5) | 555000 | * | 0.0% |
|  |  | asp424(M6) | 241000 | * | -0.1% |
|  |  | asp425(M7) | 53783 | * | 0.0% |
|  |  |  |  |  |  |
| Fumarate | C12H23O4Si2 | fum287(M0) | 3520000 | 42.6% | 44.5% |
|  |  | fum288(M1) | 1280000 | 7.3% | 5.5% |
|  |  | fum289(M2) | 1340000 | 10.9% | 11.0% |
|  |  | fum290(M3) | 1090000 | 9.9% | 9.8% |
|  |  | fum291(M4) | 2670000 | 29.2% | 29.1% |
|  |  | fum292(M5) | 567000 | * | 0.1% |
|  |  | fum293(M6) | 242428 | * | 0.1% |
|  |  | fum294(M7) | 33400 | * | 0.0% |

**Table 1: Comparison of Escher-Trace and IsoCor Stable Isotope Correction**

***No value reported**
